# Protective Effects of Niacin on Rumen Epithelial Cell Barrier Integrity in Heat-Stressed Beef Cattle

**DOI:** 10.3390/ani14020313

**Published:** 2024-01-19

**Authors:** Bicheng Zou, Fan Long, Fuguang Xue, Chuanbin Chen, Xian Zhang, Mingren Qu, Lanjiao Xu

**Affiliations:** Jiangxi Province Key Laboratory of Animal Nutrition, Engineering Research Center of Feed Development, Jiangxi Agricultural University, Nanchang 330045, China; zoubicheng1997@163.com (B.Z.); longfan9807@163.com (F.L.); xuefuguang1024@jxau.edu.cn (F.X.); chenchuanb8297@163.com (C.C.); zhangxian517@163.com (X.Z.); qumingren@jxau.edu.cn (M.Q.)

**Keywords:** niacin, heat stress, Jinjiang bulls, rumen epithelial barrier, tight junction

## Abstract

**Simple Summary:**

Heat stress can easily cause a series of physiological hazards in beef cattle, affecting the rumen barrier function and, ultimately, beef cattle performance. As a B vitamin with anti-inflammatory and anti-heat stress effects, it is essential to investigate whether niacin has a protective and ameliorative effect on the rumen barrier function in heat-stressed beef cattle. In this study, the plasma levels of IL-1β, IL-2, IL-6, and TNF-α decreased, IL-4 increased, and LPS decreased in beef cattle supplemented with niacin. These indicators are closely related to inflammation and intestinal permeability. The expression of the tight junction proteins mRNA ZO-1 and Occludin, which are closely associated with rumen barrier function, was enhanced. Histological observations showed no damage to the rumen epithelium. The results of this study suggest that niacin supplementation in heat-stressed beef cattle can reduce inflammation, improve intestinal permeability, and protect the rumen epithelial barrier function.

**Abstract:**

The present study investigates the theoretical basis for maintaining normal physiological functions in heat-stressed beef cattle by exploring the effects of niacin supplementation on the permeability of the rumen epithelial cell barrier. Herein, 12 Jinjiang bulls with an average weight of approximately 400 ± 20.0 kg were randomly divided into three groups, thermoneutral (TN), heat-stressed (HS), and heat-stressed niacin-supplemented (HN) groups, with 4 bulls in each group. The experiment spanned 70 days, and the plasma concentrations of D-lactic acid, diamine oxidase (DAO), lipopolysaccharides (LPSs), and inflammatory cytokines were analyzed. Additionally, we assessed the gene expression of tight junction proteins to understand the effect of niacin supplementation on heat-stressed beef cattle. Our results revealed that heat stress significantly increased the D-lactic acid and LPS levels in beef cattle plasma on days 30 and 45 of the experiment (*p* < 0.05). Moreover, it led to a significant rise in DAO levels on day 30 (*p* < 0.05). Niacin supplementation significantly reduced the LPS levels on day 30 (*p* < 0.05). Heat stress significantly elevated the plasma concentrations of inflammatory cytokines interleukin-1β (IL-1β), IL-2, IL-6, and tumor necrosis factor-α (TNF-α) (*p* < 0.05), while reducing the IL-4 concentration (*p* < 0.05). However, niacin supplementation effectively mitigated the concentrations of these inflammatory factors by reducing IL-1β, IL-2, IL-6, and TNF-α concentrations and increasing IL-4 concentrations. The mRNA expressions of tight junction proteins zonula occluden-1 (ZO-1), claudin-1, claudin-4, and claudin-7 were significantly downregulated (*p* < 0.05) in the HS group compared to those in the TN group, and those of ZO-1 and occludin were significantly upregulated (*p* < 0.05) in the HN group compared to those in the HS group. Notably, no significant differences were observed in ruminal papillae length and width among the studied groups (*p* > 0.05). Our findings indicate that heat stress adversely impacted the tight junction structure of the rumen epithelium, leading to a significant reduction in the expression of tight junction protein mRNA. Consequently, heat stress impaired the rumen mucosal barrier function, resulting in increased intestinal permeability. The mechanism underlying this effect may be associated with the decreased expression of tight junction protein genes in the rumen epithelial cells. However, niacin supplementation mitigated the detrimental effects of heat stress on intestinal permeability in beef cattle and increased the expression of tight junction protein genes in the rumen epithelium, thereby effectively protecting the rumen barrier in heat-stressed beef cattle. These results highlight the potential of nicotinic acid as a protective agent against the negative impacts of heat stress on intestinal integrity in beef cattle.

## 1. Introduction

Heat stress in beef cattle is known to induce oxidative stress and inflammation, leading to the impairment of the rumen epithelial barrier and an increased risk of health issues such as rumen microbial dysregulation and rumen acidosis [1]. As a consequence, the overall growth performance of the cattle may be reduced [2]. As the rumen plays a vital role in feed digestion and ruminant nutrition, maintaining the integrity of the rumen epithelial barrier function is crucial for efficient digestion. The rumen epithelium serves as a protective barrier against the luminal contents, engaging in essential functions such as nutrient absorption, pH regulation, immune defense, and overall barrier protection [3].

Heat stress disrupts the metabolism of rumen papillae and alters the rumen fermentation of the beef cattle, which subsequently affects the epithelial barrier function [4]. Micromorphological observations have suggested that heat stress exacerbates the exfoliation of the stratum corneum, leading to the destruction of the rumen epithelium’s physical barrier [4]. Therefore, ensuring the homeostasis of ruminal epithelial barrier function is crucial for the health of ruminants. The junctions between rumen epithelial cells, particularly tight junctions (TJs), are essential for maintaining the integrity of the rumen epithelium. These junctions, from the top to the basement membrane, include TJs, gap junctions, adhesion junctions, and desmosome junctions [5,6].

Niacin, a water-soluble vitamin feed additive, is widely used to reduce the incidence of ketosis during early lactation and alleviate heat stress in dairy cows [7,8]. Our previous studies have demonstrated that niacin supplementation improves the performance and rumen fermentation function of heat-stressed beef cattle, which lead to alterations in the rumen microbiota structure [9]. The rumen epithelium holds a unique position for host–microbe interactions, which profoundly impact the net nutrient utilization of the host [10,11]. There exists a direct association between rumen bacteria and the host’s capacity to efficiently absorb nutrients and maintain the integrity of the epithelial barrier in the rumen. Correlation analyses further indicate that the rumen microbiota might regulate the immunological barrier function and rumen fermentation metabolism genes, consequently affecting genes linked to rumen epithelial transcription [12,13]. These findings suggest that niacin-induced changes in the rumen epithelial barrier function might be related to its impact on the rumen microbiota.

Furthermore, previous studies conducted by Digby et al. and Gambhir et al. have suggested that niacin-induced activation of GPR109A leads to the inhibition of inflammatory cytokine and chemokine expression in epithelial cells [14,15]. It was reported that niacin had anti-inflammatory functions, potentially enhancing the function of the rumen barrier [16]. Given these findings, the present study aims to investigate the effect of niacin supplementation on the permeability of the rumen epithelial cell barrier in heat-stressed beef cattle. Our hypothesis posits that heat stress may trigger tissue inflammation and disrupt the barrier function of rumen epithelial cells, while niacin supplementation could potentially protect the rumen barrier function. To elucidate these effects, we conducted an examination of the micromorphology and gene expression patterns of rumen epithelial cells in heat-stressed beef cattle, focusing on the role of niacin supplementation.

## 2. Materials and Methods

### 2.1. Animal Ethics

The experimental procedures and protocols employed in this study were subjected to rigorous review and approval by the Committee for the Care and Use of Experimental Animals at Jiangxi Agricultural University (JXAULL-20210066).

### 2.2. Diet, Experiment Design, and Animals

The experimental trial was conducted in Gao’an, Jiangxi Province, during the summer of 2020 (July to September). Twelve Jinjiang bulls, with an average age of 24 ± 2 months and a weight of 400 ± 20.0 kg, were selected for this study. The rearing trial spanned 70 days, including a 10-day acclimatization period followed by a 60-day experimental period. The animals were randomly assigned to three treatment groups: thermoneutral (TN), heat-stressed (HS), and heat-stressed niacin-supplemented (HN) groups. In the TN group, the beef cattle were transported to a climate-controlled chamber and maintained under thermoneutral conditions, with a temperature of 26 ± 2 °C and humidity of 30 ± 2.7%. They were fed a basal diet during the experiment and were in a state of non-heat stress [mean temperature–humidity index (THI) = 71.10 ± 0.62%]. Beef cattle in the HS and HN groups were housed in the same high-temperature, high-humidity barn during the summer months of July through September, and both experienced heat stress, with an average daily temperature of 31.55 ± 2.61 °C, an average daily humidity of 66.66 ± 8.66%, and THI > 72.The HS group was fed a basal diet. And in the HN group, 800 mg/kg of niacin (DM basis) was added to the concentrate, and during the preparation process, niacin was first added to the premix and mixed well and then mixed step by step to form the concentrate (Jiangxi Brother Medicine Co., Ltd., Nanchang, China). All animals were fed twice daily at 6:00 and 16:00. They had constant access to freshwater through automatic waterers. The basal diets and nutritional group compositions are detailed in Table 1.

### 2.3. Temperature–Humidity Index Measurement

During the trial, measurements of relative humidity and temperature inside the barns of cattle in the HS and HN groups were taken at 07:30, 13:30, and 19:30 each day. Subsequently, the average daily temperature, average relative humidity, and average daily THI were calculated. The THI was calculated using the following formula:

THI = (1.8 × T + 32) − [(0.55 − 0.0055 × RH) × (1.8T − 26.8)],

where T represents the dry-bulb temperature (°C), and RH denotes the relative humidity (%) [17].

### 2.4. Sample Collection and Analysis

Blood samples were collected on the specified sampling days, that is, days 1, 15, 30, 45, and 60, and beef cattle fasted for 12 h before sampling. The samples were obtained via the jugular vein and collected into 5 mL vacuum tubes containing sodium heparin. Subsequently, they were subjected to centrifugation at 3500× *g* for 15 min at 4 °C to separate the plasma. The separated plasma was then preserved at −20 °C for subsequent analysis.

### 2.5. Intestinal Permeability Assay

To assess intestinal permeability, the levels of D-lactic acid, diamine oxidase (DAO), and lipopolysaccharide (LPS) in the plasma were measured. For this purpose, commercially available enzyme-linked immunosorbent assay (ELISA) kits from Shanghai Enzyme Link Biotechnology Co., Ltd. (Shanghai, China) were utilized.

### 2.6. Inflammatory Cytokines Assays

The concentrations of key inflammatory cytokines interleukin-1 (IL-1), IL-2, IL-4, IL-6, and tumor necrosis factor-α (TNF-α) in the plasma were evaluated using commercially available radioimmunoassay kits (the Beijing Huaying Institute of Biotechnology, Beijing, China).

### 2.7. Histologic Analysis

On day 60 of the trial, following a 12 h fast, all 12 Jinjiang cattle were slaughtered using the captive bolt knockout method, adhering to national Standard Operating Procedures (GB/T 19477-2004, China [18]). Subsequently, ruminal tissue samples were taken after washing with phosphate-buffered saline. Rumen tissue samples with a total thickness of 1~3 cm were removed from the ventral sac and preserved in a 4% paraformaldehyde solution for histological examination. Additionally, 10 g of rumen epithelium was obtained from the ventral sac site of the rumen, snap-frozen in liquid nitrogen, and stored at −80 °C for subsequent RNA extraction.

The tissue samples were sectioned into 1 × 1 cm pieces and placed in a 4% paraformaldehyde solution for histomorphological analysis. The morphology of the rumen epithelium was examined using hematoxylin and eosin (H&E) staining. After paraffin embedding, the samples were cut into 5 μm sections on the longitudinal plane and mounted on slides. The slides were stained with H&E using conventional methods. The sections were examined under a DM3000 microscope (Leica, Wetzlar, Germany) with three replicates per sample. The height and width of the papillae were measured using the Image-Pro program. Six papillae were randomly selected for each sample, and the average value was calculated.

### 2.8. Quantitative Real-Time Polymerase Chain Reaction

Total RNA was extracted from rumen tissue samples using the TransZol UP Plus RNA Kit (TransGen Biotech, Beijing, China) following the manufacturer’s instructions. The integrity and purity of RNA were detected by gel electrophoresis. And the purity and concentration of RNA samples were determined with a NanoDrop ND-2000 1 spectrophotometer (Thermo Fisher Scientific Inc., Waltham, MA, USA) to ensure that the ratio of A260/A280 nm ratio was between 1.8 and 2.0. Total RNA (1 μg) was then reverse-transcribed into cDNA in a final volume of 20 μL using the cDNA Synthesis SuperMix kit as per the manufacturer’s instructions. Primers used for the quantitative real-time polymerase chain reaction (PCR) were provided by Shanghai Jierui Bioengineering Co., Ltd. (Shanghai, China) and are listed in Table 2. β-actin was used as an internal reference. The quantitative real-time PCR was performed using the PerfectStartTM Green qPCR SuperMix kit, following the manufacturer’s directions, using a CFX Connect Real-Time PCR Detection System (Bio-Rad, Hercules, CA, USA). The following methodology was used for the PCR amplification: 42 cycles at 94 °C for 30 s, 5 s, and 62 °C for 30 s of annealing. The data from the quantitative PCR were analyzed using the 2^−∆∆Ct^ method.

### 2.9. Statistical Analyses

Statistical analyses were performed using SPSS 23.0 software (IBM Corp, Armonk, NY, USA). Firstly, all experiment data were tested for normal distribution, after which independent samples *t*-tests were conducted to compare the TN and HS groups as well as the HS and HN groups. A significance level of *p* < 0.05 was considered as indicating a significant difference. The various indicators of intestinal mucosal permeability (D-lactate, DAO, and LPS), inflammatory cytokines, TJ protein gene expression, and rumen epithelial tissue were analyzed using these statistical tests.

## 3. Results

### 3.1. Subsection

#### 3.1.1. Temperature-Humidity Index

**Figure 1:**
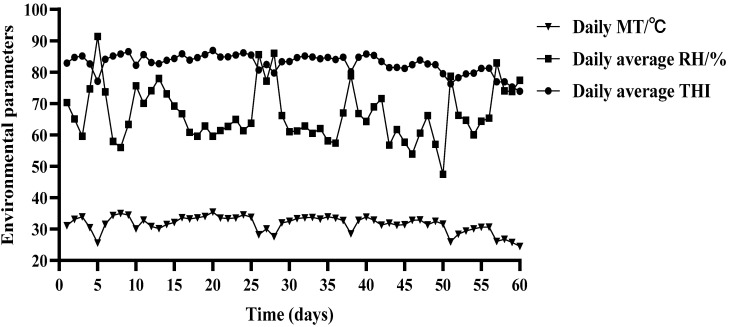
Average daily temperature, relative humidity, and THI of the barn in the HS and HN groups.

At present, THI is the commonly used index to evaluate the degree of heat stress in livestock and poultry; when THI > 72, the animal is in a state of heat stress, of which, 72 < THI < 78 is mild heat stress, 78 < THI < 88 is moderate heat stress, and THI > 88 is severe heat stress. As depicted in Figure 1, over the entire duration of the experiment, the average daily temperature of the barn housing the test beef cattle from the HS and HN groups was 31.55 °C, with an average daily humidity of 66.66%. The average daily THI recorded was 82.74, encompassing the highest and lowest THI values of 89.89 and 73.88, respectively. Notably, 54 days fell within the THI range of 78–88, indicating that the test beef cattle in both groups experienced thermal stress conditions.

#### 3.1.2. Intestinal Permeability

As shown in Table 3, compared to those in the TN group, the D-lactate levels in beef cattle within the HS group were significantly higher on days 30 and 45 (*p* < 0.05). Concurrently, on day 30, the levels of DAO in beef cattle from the HS group were significantly elevated in comparison to those in the TN group (*p* < 0.05). No significant differences in D-lactate and DAO levels were observed between the HS and HN groups. Moreover, the LPS levels were significantly elevated in the HS group than those in the TN group on days 30 and 45 (*p* < 0.05). Notably, the LPS levels were significantly lower in the HN group than those in the HS group on day 30 (*p* < 0.05).

#### 3.1.3. Plasma Cytokine Profile

As shown in Table 4, compared with the TN group, plasma IL-1β, IL-2, IL-6, and TNF-α levels in the HS group were significantly increased on days 1, 15, 30, 45, and 60 (*p* < 0.05), and plasma IL-4 levels in the HS group were significantly decreased on days 1, 15, 30, and 45 (*p* < 0.05). Furthermore, compared with the HS group, IL-1 β levels in the HN group were significantly decreased on days 1 and 15 (*p* < 0. 05), IL-2 levels were significantly decreased on days 1, 15, 30, and 60 (*p* < 0. 05), IL-4 levels were significantly increased on days 15, 30, and 45 (*p* < 0.05), IL-6 levels were significantly decreased on days 1 and 45 (*p* < 0.05), and TNF-α levels were significantly decreased on days 15, 30, 45, and 60 (*p* < 0.05). Collectively, the impact of heat stress resulted in elevated plasma levels of IL-1β, IL-2, IL-6, and TNF-α, along with decreased levels of IL-4. Notably, niacin supplementation led to decreased plasma levels of IL-1β, IL-2, IL-6, and TNF-α and increased levels of IL-4 in heat-stressed beef cattle.

#### 3.1.4. The Expression and Histological Analysis of Tight Junction Protein-Related Genes in Rumen Epithelium

**Figure 2:**
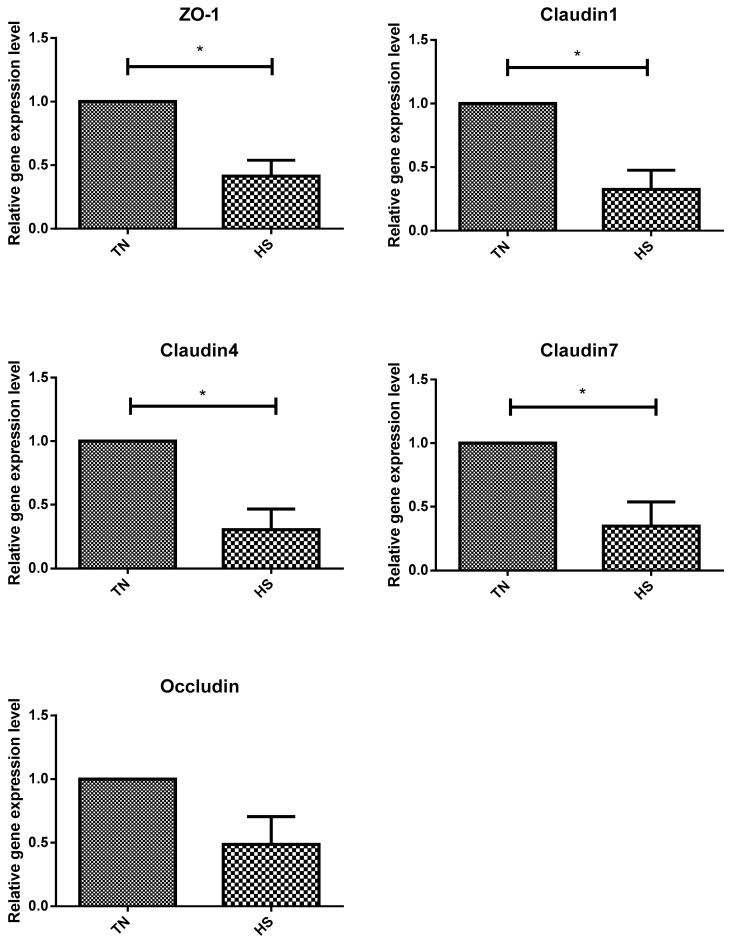
The effect of heat stress on the expression of tight junction protein gene in beef cattle. TN = thermoneutral group. HS = heat stress group. An “*” indicates a significant difference (*p* < 0.05).

**Figure 3:**
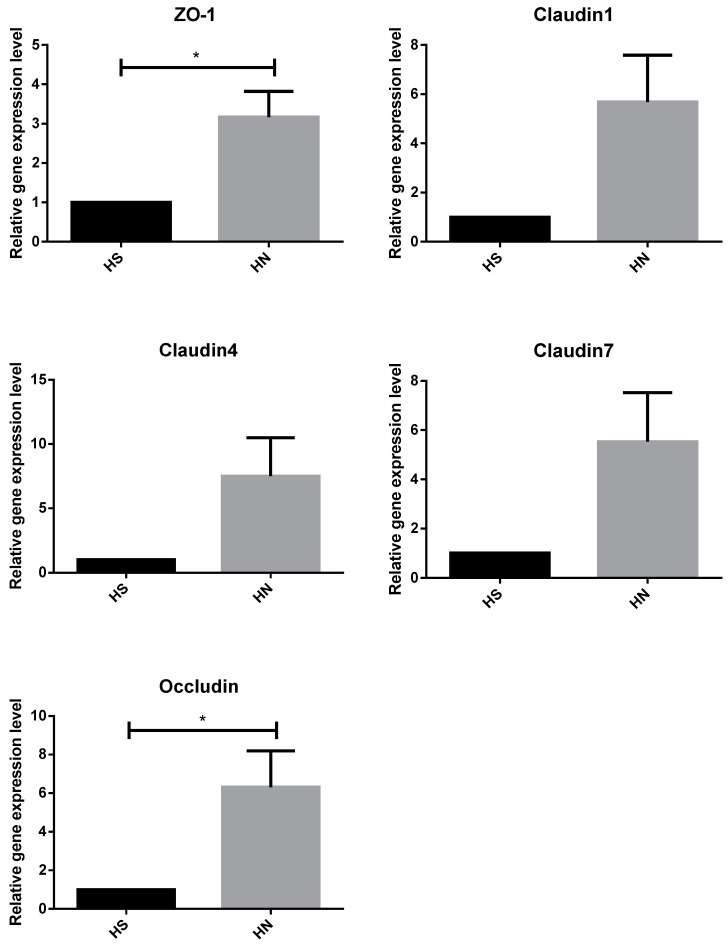
Effects of niacin on the expression of tight junction protein gene in heat-stressed beef cattle. HS = heat stress group. HN = heat stress supplemented with niacin treatment group. An “*” indicates a significant difference.

**Figure 4:**
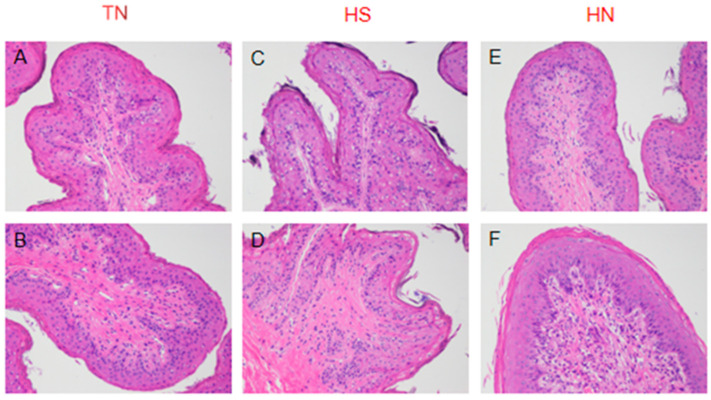
Representative histology sections of the rumen papillae of beef cattle from the thermoneutral group (TN), heat stress group (HS), and heat stress supplemented with niacin group (HN). Representative micrographs of H&E staining are shown (4 × 100 multiple microscope). (**A**,**B**) belong to the TN group. (**C**,**D**) belong to the HS group. (**E**,**F**) belong to the HN group.

The relative mRNA expressions of TJ proteins zonula occluden-1 (ZO-1), claudin-1, claudin-4, and claudin-7 were significantly lower in the HS group than in the TN group (*p* < 0.05). However, no significant differences were observed in the relative expression of the occludin gene between the TN and HS groups (Figure 2). The relative expression of the TJ protein ZO-1 and occludin genes was significantly higher in rumen epithelial cells of the HN group cattle than that in those of the HS group (*p* < 0.05). Notably, no significant difference was observed in the relative expression of claudin-1, claudin-4, and claudin-7 genes between the HS and HN groups (Figure 3). In terms of histological observations, the TN group cattle displayed an intact corneum layer (Figure 4A,B). In contrast, the corneum layer of the HS group cattle exhibited signs of damage and shedding (Figure 4C,D). The HN group cattle displayed a minor lesion compared to the cuticle (Figure 4E,F). Notably, as shown in Table 5, no significant differences were observed in ruminal papillae length and width among the treatment groups.

## 4. Discussion

### 4.1. Effects of Niacin on Intestinal Permeability and Inflammatory Cytokines in Heat-Stressed Beef Cattle

The preservation of the integrity of the ruminal epithelial barrier function holds significant importance in upholding ruminant gut immunity and overall body health. The rumen serves as a semipermeable paracellular diffusion barrier that plays a multifaceted role in various biological functions, encompassing the modulation of nutrient absorption and the mitigation of immunoreactions [19]. Maintaining the rumen homeostasis is crucial, as disruptions such as heightened release of bacterial LPSs can compromise the functionality of the ruminal epithelial barrier. The absorptive function of the rumen must be highly selective to prevent the simultaneous influx of microorganisms and toxins from the rumen into the bloodstream [20]. Research indicates a direct correlation between the host’s inflammatory response and the presence of specific aberrant metabolites in the rumen, particularly LPS [21]. The presence of LPS in the rumen could potentially augment permeability of the rumen wall, thereby impacting the structural integrity of the rumen epithelium and potentially impairing its barrier function [22]. LPS and the substantial release of inflammatory factors can affect the TJs of rumen epithelial cells, impair the barrier function of rumen epithelial cells, and result in elevated permeability of the rumen mucosa [23].

Elevated LPS concentrations within the systemic circulation can serve as a reliable marker, indicating the potential severity of damage to the integrity of the intestinal mucosal barrier [24]. Our findings reveal that LPS plasma levels were significantly higher in beef cattle from the HS group than in those from the TN group. Notably, a significant difference was observed on days 30 and 45 of the experimental period, respectively. These findings indicate a progressive increase in LPS levels with an increase in the duration of heat stress, contributing to heightened intestinal permeability and a discernible impairment of the rumen barrier function. The supplementation of niacin introduced a mitigating effect on LPS levels, suggesting a potential safeguarding role of niacin against increased intestinal permeability in heat-stressed beef cattle.

Heat stress elicits an enhanced expression of pro-inflammatory cytokines and a concomitant reduction in anti-inflammatory cytokines in broilers [25]. Existing research underscores the intrinsic relationship between ruminal epithelial inflammation and the consequential disruption of barrier structure and function [26]. Feng et al. revealed that niacin supplementation effectively downregulates the expression of TNF-α, IL-8, γ-interferon (IFN-γ), and IL-1β [27]. The increase in the concentration of LPS within the systemic circulation serves as a potential indicator signifying the development of a severe injury to the integrity of the intestinal mucosal barrier. Herein, heat stress induced a significant increase in the levels of specific inflammatory cytokines IL-1β, IL-2, IL-6, and TNF-α in the bloodstream of beef cattle, and the release of these pro-inflammatory factors was associated with LPS-induced activation of NF-κB and MAPK pathways, indicating that heat stress not only causes an increase in the intestinal permeability but also induces a systemic inflammatory response. However, niacin supplementation caused a significant decrease in the levels of these indicators. This aligns with the findings of Feng et al. [27], confirming niacin’s potential to ease inflammation in heat-stressed cattle. Niacin likely relieves the inflammatory damage caused by heat stress in beef cattle by reducing the levels of pro-inflammatory factors IL-1β, IL-2, IL-6, and TNF-α, while boosting the level of the anti-inflammatory factor IL-4.

### 4.2. Effects of Niacin on Gene Expression and Tissue Structure of Tight Junction Protein in Heat-Stressed Beef Cattle

It has been well-established in scientific literature that disruptions in rumen metabolism can trigger the release of bacterial LPSs or other harmful substances, thereby compromising the integrity of rumen epithelial cells and the intercellular TJs. Consequently, there is a subsequent reduction in the absorption rate of nutrients across the epithelial lining, an elevation in permeability, impairment of barrier functionality, and an increased translocation of bacterial toxins into the bloodstream. These interconnected events can trigger systemic pathological responses, ultimately causing a significant impact on animal productivity. Animals subjected to heat stress exhibit compromised intestinal health, accompanied by a malfunction of the intestinal barrier [28,29,30], which is consistent with the findings of the present study. Our study substantiates that heat stress may increase intestinal permeability, exerting a detrimental effect on the intestinal health of beef cattle. This assertion is supported by our assessments of both the intestinal permeability index such as D-lactic acid, DAO, and LPS, and the levels of plasma inflammatory cytokines.

The findings of the present study unveiled a substantial reduction in the mRNA relative expression levels of TJ proteins ZO-1, claudin-1, claudin-4, and claudin-7 in rumen epithelial cells under the influence of heat stress. The significance of direct cellular interactions mediated by TJs is paramount in establishing selective barriers. These TJs are constituted by a complex interplay of integral transmembrane proteins, notably claudins and occludins, along with the adapter proteins ZO-1, ZO-2, and ZO-3 [31,32,33]. The expression of occludin has been correlated with a spectrum of junctional functions, revealing that augmenting occludin expression enhances barrier function and transmembrane resistance in mammalian epithelial cells [34,35].

Moreover, claudins play an indispensable role in upholding ion permeability and preserving the barrier function at the intercellular junctions [36,37]. Previous research has demonstrated that the overexpression of claudin-1 leads to the enhancement of the barrier function [38]. Notably, ZO-1 is a crucial TJ component that engages in critical interactions with both occludins and claudins, underscoring its pivotal role [39]. Therefore, it is plausible to hypothesize that the effect of heat stress on the rumen epithelium could have incited a responsive mechanism, potentially downregulating the expression of closely interrelated genes within the cellular framework and thereby disrupting the rumen barrier’s functionality. Herein, the significant upregulation in the expression of ZO-1 and occludin genes after niacin supplementation suggests a reinforcing effect of niacin on the protective function of the rumen barrier. These positive effects could potentially stem from the enhancement of rumen epithelium metabolism after niacin supplementation.

Our findings from the micromorphological analysis that heat stress aggravates cuticle shedding and contributes to the disruption of the physical barrier of the rumen epithelium are consistent with those of a previous study [4]. Heat stress caused detrimental alterations to the tissue structure of the rumen epithelium and particularly resulted in cuticle degradation. Such disruptions could facilitate the entry of pathogenic bacteria and toxic substances into the body through the compromised rumen wall, consequently impinging on physiological processes. This phenomenon is likely underpinned by the downregulation of ZO-1, claudin-1, and occludin expression in the rumen epithelium. However, niacin supplementation demonstrated a mitigating effect on this condition, which could be attributed to its potential to attenuate the inflammatory response and upregulate TJ protein gene expression.

## 5. Conclusions

In conclusion, this study underscores that heat stress increases intestinal permeability, disrupts rumen epithelial cell integrity and barrier function, and influences the relative expression of TJ genes. Niacin supplementation emerges as a potential remedy to counteract these heat-induced effects. By attenuating the damage to rumen epithelium caused by heat stress and augmenting the relative expression of TJ protein-related genes, niacin supplementation maintains the integrity of the rumen epithelial barrier function. This intervention holds promise for improving the health and productive performances of livestock under conditions of heat stress.

## Figures and Tables

**Table 1:** Composition and nutrient levels of the basal diet (air dry basis).

Items	Content (%)
Ingredients,%	
Brewers grains	20.00
Rice straw	20.00
Corn	46.80
Soybean meal	9.00
Premix ^a^	2.40
NaHCO_3_	1.20
NaCl	0.60
Total	100.00
Nutrient composition,%	
DM	95.67
CP	14.44
EE	4.03
Ash	8.31
NDF	26.99
ADF	11.62
Ca	0.69
P	0.36

^a^ One kilogram of premix provided the following: vitamin A 150,000 IU, vitamin D3 20,000 IU, vitamin E 3000 IU, Fe 3200 mg, Mn 1500 mg, Zn 2000 mg, Cu 650 mg, I 35 mg, Se10 mg, Co 10 mg, Ca 130 g, P 30 g.

**Table 2:** Information of target genes and primers.

Genes	Accession No.	Primer Sequences (5′ to 3′ Direction)
ZO-1	XM_024982002.1	Forward: TCTCGAAGATAGCCCTGCTG Reverse: AGGTCAAGCAGGAAGAGGAC
Claudin-1	NM_001001854.2	Forward: CTTCATCCTGGCGTTTCTGG Reverse: AGACTTTGCACTGGATCTGC
Claudin-4	XM_027527588.1	Forward: GCAACGACAAGCCCTACTC Reverse: AGCTCAGTCCAGGGAGAAAC
Claudin-7	XM_027519738.1	Forward: ATTCTGAAGGCGGAAATGGC Reverse: GGGCGGTGATGATATTGTCG
Occludin	NM_001082433.2	Forward: AGATGCACGTTCGACCAATG Reverse: ATTACTCCGGGAGGAGAGGT
β-actin	NM_173979.3	Forward: CCCTGGAGAAGAGCTACGAG Reverse: CAGGAAGGAAGGCTGGAAGA

**Table 3:** Effects of niacin on plasma D-lactic acid, DAO, and LPS levels in heat-stressed beef cattle.

Item		TN	HS	HN	*p*-Value	
					TN vs. HS	HS vs. HN
D-lactic acid (umol/mL)	Day 1	15.77 ± 1.88	16.68 ± 2.08	15.41 ± 2.19	0.756	0.906
	Day 15	13.61 ± 2.33	13.66 ± 2.36	12.66 ± 2.07	0.990	0.760
	Day 30	18.69 ± 1.19	23.85 ± 0.92	22.09 ± 0.86	0.014	0.213
	Day 45	16.93 ± 0.69	22.13 ± 1.00	19.67 ± 0.67	0.005	0.086
	Day 60	16.8 ± 1.67	18.03 ± 1.03	16.14 ± 0.20	0.340	0.124
DAO (ng/mL)	Day 1	5.83 ± 1.28	6.71 ± 0.84	6.62 ± 0.95	0.586	0.945
	Day 15	5.93 ± 1.28	6.39 ± 0.3	6.04 ± 0.9	0.617	0.535
	Day 30	7.86 ± 0.29	10.4 ± 0.76	9.33 ± 0.63	0.020	0.260
	Day 45	8.38 ± 0.62	10.13 ± 0.73	9.32 ± 0.42	0.117	0.788
	Day 60	7.45 ± 1.39	8.31 ± 0.79	8.2 ± 0.84	0.609	0.936
LPS (EU/L)	Day 1	12.14 ± 1.90	15.62 ± 1.79	12.78 ± 1.25	0.231	0.241
	Day 15	14.97 ± 1.21	18.85 ± 1.46	16.91 ± 1.58	0.087	0.402
	Day 30	15.44 ± 2.22	24.91 ± 2.22	15.83 ± 0.71	0.013	0.020
	Day 45	13.73 ± 1.50	27.31 ± 2.17	22.76 ± 2.10	0.002	0.125
	Day 60	13.51 ± 1.88	18.67 ± 2.01	17.04 ± 2.02	0.110	0.589

Note: The results are presented as the mean values and standard error. TN = thermoneutral treatment group; HS = heat stress treatment group; HN = heat stress supplemented with niacin treatment group.

**Table 4:** Effects of niacin on plasma inflammatory cytokines in heat-stressed beef cattle.

Item (pg/mL)		TN	HS	HN	*p*-Value	
					TN vs. HS	HS vs. HN
IL-1β	Day 1	16.40 ± 1.52	27.46 ± 0.87	23.63 ± 0.83	0.001	0.019
	Day 15	18.17 ± 1.22	27.84 ± 1.80	21.86 ± 1.28	0.004	0.035
	Day 30	16.48 ± 1.26	26.06 ± 1.83	21.19 ± 1.54	0.005	0.087
	Day 45	17.13 ± 2.12	27.42 ± 1.80	21.66 ± 1.63	0.01	0.056
	Day 60	16.32 ± 1.52	26.76 ± 1.84	21.83 ± 1.13	0.005	0.063
IL-2	Day 1	176.57 ± 19.55	313.21 ± 9.53	269.14 ± 8.02	0.001	0.012
	Day 15	189.65 ± 22.12	314.54 ± 21.81	239.82 ± 5.53	0.007	0.016
	Day 30	183.95 ± 17.15	298.66 ± 22.10	231.74 ± 14.15	0.006	0.043
	Day 45	188.20 ± 25.47	314.70 ± 20.99	247.27 ± 23.21	0.009	0.075
	Day 60	206.69 ± 31.41	314.63 ± 18.15	256.18 ± 13.96	0.025	0.043
IL-4	Day 1	10.76 ± 0.87	6.25 ± 0.22	6.68 ± 0.39	0.002	0.369
	Day 15	10.19 ± 0.96	6.21 ± 0.45	8.31 ± 0.64	0.01	0.036
	Day 30	10.35 ± 1.13	6.68 ± 0.52	8.79 ± 0.28	0.026	0.012
	Day 45	10.54 ± 1.39	6.31 ± 0.43	8.33 ± 0.51	0.027	0.022
	Day 60	10.21 ± 1.54	6.48 ± 0.43	7.68 ± 0.39	0.059	0.085
IL-6	Day 1	105.27 ± 7.19	163.80 ± 5.03	139.81 ± 3.94	0.001	0.009
	Day 15	110.06 ± 4.69	166.25 ± 10.49	135.93 ± 6.89	0.003	0.052
	Day 30	111.78 ± 8.55	155.99 ± 10.43	128.08 ± 7.74	0.017	0.075
	Day 45	109.33 ± 9.64	163.76 ± 10.53	127.14 ± 6.73	0.009	0.026
	Day 60	113.20 ± 14.36	159.93 ± 10.88	136.11 ± 6.03	0.041	0.104
TNF-α	Day 1	44.20 ± 3.94	71.76 ± 3.33	64.84 ± 1.82	0.002	0.118
	Day 15	49.37 ± 3.80	75.35 ± 5.07	59.19 ± 2.88	0.006	0.032
	Day 30	43.45 ± 2.80	70.34 ± 5.08	55.25 ± 2.84	0.004	0.041
	Day 45	46.35 ± 5.34	74.13 ± 5.02	56.06 ± 3.45	0.009	0.025
	Day 60	49.94 ± 6.73	74.82 ± 4.25	60.68 ± 3.04	0.020	0.035

Note: The results are presented as the mean values and standard error. TN = thermoneutral treatment group; HS = heat stress treatment group; HN = heat stress supplemented with niacin treatment group.

**Table 5:** Effects of niacin on length and width of rumen papillae in heat-stressed beef cattle.

Item	TN	HS	HN	*p*-Value	
				TN vs. HS	HS vs. HN
Rumen papillae length (μm)	1599.07 ± 118.56	1482.88 ± 166.96	1561.53 ± 210.01	0.591	0.779
Rumen papillae width (μm)	405.53 ± 61.33	559.32 ± 98.65	392.96 ± 42.67	0.234	0.173

Note: The result is presented as the mean values and standard error. TN = thermoneutral treatment group; HS = heat stress treatment group; HN = heat stress supplemented with niacin treatment group.

## Data Availability

The data presented in this study are available in [Protective Effects of Niacin on Rumen Epithelial Cell Barrier Integrity in Heat-Stressed Beef Cattle].
